# Targeting Cytokine Storm in COVID-19: What Have We Learned?

**DOI:** 10.1093/ehjopen/oeab005

**Published:** 2021-07-29

**Authors:** Paul M Ridker

**Affiliations:** From the Center for Cardiovascular Disease Prevention, Brigham and Women’s Hospital, Harvard Medical School, Boston, MA

## Abstract

Invited Commentary: Cremer et al, Double-Blind Randomised Proof-of-Concept Trial of Canakinumab in Patients with COVID-19 Associated Cardiac Injury and Heightened Inflammation, Eur Heart J Open (in press 2021)

Word Count: 1100 References: 10

Short Title: Targeting Cytokines in COVID-19

pridker@bwh.harvard.edu tel 617-759-0716 fax 617-734-1508

Disproportionate immune activation in the form of cytokine release syndrome is a major determinant of poor outcome among critically ill patients with COVID-19 disease. As a consequence, elevated levels of several pro-inflammatory cytokines (interleukin-1, interleukin-6, tumor necrosis factor $\alpha$), acute phase reactants (C-reactive protein, ferritin), and coagulation biomarkers (D-dimer) all associate with disease severity[1]. During the pandemic, multiple randomized clinical trials have been conducted to address whether dampening the innate immune response might improve patient outcomes among those with inflammation-mediated lung injury.

Beyond vaccine development and the use of prone positioning for ventilated patients, the most important disease-specific intervention for COVID-19 has proven to be the use of corticosteroids. Within the efficient and groundbreaking RECOVERY platform trial conducted within the United Kingdom, the use of 6 mg dexamethasone daily for up to 10 days resulted in a 17 percent reduction in mortality when compared to open control (rate ratio 0.83, 95%CI 0.75-0.93, P<0.0001)[2]. This benefit was almost entirely observed among those receiving mechanical ventilation (rate ratio 0.64, 95%CI 0.51-0.81) and among those receiving oxygen without invasive ventilation (rate ratio 0.82, 95%CI 0.72-0.94), but not among patients with less severe pulmonary compromise (rate ratio 1.19, 95CI 0.92-1.55). Based upon these data, severely compromised COVID-19 patients worldwide immediately began to receive high dose corticosteroids with considerable benefit.

The central signaling cytokine interleukin-6 has also proven to be a target for intervention in COVID-19 disease. However, unlike the situation with steroids, the use of interleukin-6 inhibitors evolved from case reports and small case series which then led to small, moderate, and ultimately large definitive clinical trials. Most of this work was been done with tocilizumab, a recombinant monoclonal antibody that inhibits binding of interleukin-6 to membrane as well as soluble interleukin-6 receptors.

It is important to recognize that results for tocilizumab were mixed even among the moderate sized randomized trials, due partly to limited power and partly to differential enrollment criteria and different levels of illness severity. For example, among 482 patients hospitalized with severe COVID-19 pneumonia randomly allocated to a single infusion of tocilizumab or placebo in the COVACTA trial, active therapy neither improved clinical status (P=0.31) nor reduced mortality (19.7% versus 19.4 %, P = 0.94)[3]. Similarly, the BACC Bay Tocilizumab Trial did not demonstrate efficacy of tocilizumab for preventing intubation or death among 243 moderately ill hospitalized COVID-19 patients[4]. An intermediate result was reported in the EMPACTA trial of 377 patients hospitalized with COVID-19 pneumonia who did not require mechanical ventilation at the time of enrollment; in that setting, tocilizumab reduced the likelihood of a composite endpoint of progressing to mechanical ventilation or death, but did not lower all-cause mortality (10.4% on tocilizumab, 8.6% on placebo)[5].

By contrast, in the REMAP-CAP trial inclusive of 803 critically ill ICU patients, a pooled analysis of those allocated to tocilizumab or to a second interleukin-6 inhibitor, sarilumab, showed a modest but statistically significant survival benefit[6]. Yet, it took a far larger randomized trial to change medical practice; in a second arm of the RECOVERY platform trial involving 4,116 acutely ill adults with COVID-19 disease, hypoxia and CRP levels >75mg/L, tocilizumab clearly reduced mortality as compared to usual care by 15 percent (rate ratio 0.85, 95%CI 0.76-0.94, P = 0.003), including among those already treated with corticosteroids[7]. Further, within RECOVERY, those allocated to tocilizumab were more likely to be discharged from hospital within 28 days and were less likely to require mechanical ventilation (both P-

values < 0.0001). This series of trials successfully demonstrates the evolution of scientific investigation as it moves from small hypothesis generating studies to definitive outcome trials.

Beyond steroids and interleukin-6, it is logical to look upstream in the canonical interleukin-1 to interleukin-6 pathway of innate immunity for additional COVID-19 treatment targets. In parallel with the case for interleukin-6 inhibition, this journey has of necessity started small. In this issue of *European Heart Journal Open*, Cremer and colleagues in the Three C Study present an hypothesis generating trial comparing canakinumab 300mg, canakinumab 600mg, and placebo in the setting of hospitalized COVID-19 patients with elevated troponins and evidence of major systemic inflammation (CRP >50mg/L)[8]. Canakinumab is the same fully human monoclonal targeting inerleukin-1β that showed efficacy for cardiovascular event reduction in the CANTOS trial[9].

Of the 45 participants randomized in the Three C Study, 60 percent also received corticosteroids and/or the anti-viral remdesivir either at enrollment or during the index hospitalization. As reported, there was no difference in the trial primary endpoint of time to clinical improvement when comparing either dose of canakinumab to placebo. Similar rates of clinical recovery at day 14 were observed across treatment groups with none of the patients who were intubated at study entry demonstrating clinical improvement according to trial group. Although total exposure was limited, no safety issues were uncovered.

Cremer et al additionally note in secondary analyses that by day 28, patients who received 600 mg IV canakinumab were numerically somewhat more likely to show clinical improvement, but are appropriately cautious in pointing out that this is both a *post-hoc* and non-significant analysis based on a very small number of outcomes. Hence, these preliminary data for interleukin-1β inhibition among COVID patients could initiate the same scientific cycle that resulted in clarity regarding interleukin-6 inhibition, as described above. There is no guarantee of success with anti-inflammatory therapy in the setting of COVID-19; this is perhaps best demonstrated in the 4,488 participant COLCORONA trial evaluating the anti-inflammatory colchicine in an outpatient setting[10]. While COLCORONA is a remarkable logistic achievement, the data show no significant benefit for the primary trial endpoint of death or hospitalization for COVID-19 (odds ratio 0·79, 95·1% CI 0·61–1·03, P=0·081). COLCORONA also reported an unanticipated increase in pulmonary embolism (11 on colchicine, 2 on placebo) though none of these resulted in need for mechanical ventilation or death, and pro-thrombotic effects of colchicine have not been observed in other settings.

When the history of COVID-19 pandemic is finally written, all of these clinical trials - small and large, as well as efforts addressing anticoagulants, anti-thrombotics, convalescent plasma, and neutralizing monoclonal antibodies - will be seen as important steps in the global medical community's attempts to address a crisis of staggering proportion. We are extraordinarily lucky to have multiple vaccines that vastly reduce the need for any of these therapeutic interventions. Thus, a major lesson learned when we face an inevitable recurrent pandemic is that patient education and the social structures to overcome vaccine hesitancy need to be addressed up front. Regrettably, solutions to these very human issues are neither as easily studied nor as easily implemented as are results from randomized controlled trials.
